# From early-onset asthma to chronic obstructive pulmonary disease: potential mediating proteins and therapeutic targets

**DOI:** 10.1093/bib/bbag209

**Published:** 2026-05-06

**Authors:** Yuhan Jiang, Ju Guo, Yifan Wang, Run Guo, Yongjian Wei, Tianchun Li, Xuelin Wang, Ruiwen Xia, Wanyi Li, Yingxue Zou, Hongxi Yang

**Affiliations:** Department of Pulmonology, Children’s Hospital, Tianjin University/Tianjin Children’s Hospital, No. 225 Machang Road, Hexi District, Tianjin 300202, China; Tianjin Key Laboratory of Birth Defects for Prevention and Treatment, No. 238 Longyan Road, Beichen District, Tianjin 300074, China; Clinical School of Pediatrics, Tianjin Medical University, No. 22 Qixiangtai Road, Heping District, Tianjin 300070, China; Department of Ophthalmology, Tianjin Medical University General Hospital, No. 154 Anshan Road, Heping District, Tianjin 300052, China; Department of Rheumatology and Immunology, West China Hospital, Sichuan University, No. 37 Guoxue Lane, Wuhou District, Chengdu 610041, Sichuan, China; Department of Pulmonology, Children’s Hospital, Tianjin University/Tianjin Children’s Hospital, No. 225 Machang Road, Hexi District, Tianjin 300202, China; Tianjin Key Laboratory of Birth Defects for Prevention and Treatment, No. 238 Longyan Road, Beichen District, Tianjin 300074, China; Province and Ministry Co-sponsored Collaborative Innovation Center for Medical Epigenetics, State Key Laboratory of Experimental Hematology, School of Basic Medical Sciences, Tianjin Medical University, No. 22 Qixiangtai Road, Heping District, Tianjin 300070, China; Chinese Academy of Medical Sciences and Peking Union Medical College, No. 1 Shuaifuyuan, Dongdan, Dongcheng District, Beijing 100730, China; Department of Pulmonology, Children’s Hospital, Tianjin University/Tianjin Children’s Hospital, No. 225 Machang Road, Hexi District, Tianjin 300202, China; Tianjin Key Laboratory of Birth Defects for Prevention and Treatment, No. 238 Longyan Road, Beichen District, Tianjin 300074, China; Department of Pulmonology, Children’s Hospital, Tianjin University/Tianjin Children’s Hospital, No. 225 Machang Road, Hexi District, Tianjin 300202, China; Tianjin Key Laboratory of Birth Defects for Prevention and Treatment, No. 238 Longyan Road, Beichen District, Tianjin 300074, China; Clinical School of Pediatrics, Tianjin Medical University, No. 22 Qixiangtai Road, Heping District, Tianjin 300070, China; Department of Pulmonology, Children’s Hospital, Tianjin University/Tianjin Children’s Hospital, No. 225 Machang Road, Hexi District, Tianjin 300202, China; Tianjin Key Laboratory of Birth Defects for Prevention and Treatment, No. 238 Longyan Road, Beichen District, Tianjin 300074, China; Department of Pulmonology, Children’s Hospital, Tianjin University/Tianjin Children’s Hospital, No. 225 Machang Road, Hexi District, Tianjin 300202, China; Tianjin Key Laboratory of Birth Defects for Prevention and Treatment, No. 238 Longyan Road, Beichen District, Tianjin 300074, China; Clinical School of Pediatrics, Tianjin Medical University, No. 22 Qixiangtai Road, Heping District, Tianjin 300070, China; Province and Ministry Co-sponsored Collaborative Innovation Center for Medical Epigenetics, State Key Laboratory of Experimental Hematology, School of Basic Medical Sciences, Tianjin Medical University, No. 22 Qixiangtai Road, Heping District, Tianjin 300070, China

**Keywords:** chronic obstructive pulmonary disease, asthma, drug target, mediating protein

## Abstract

**Background and objective:**

Early-onset asthma (EOA) significantly increases the risk of chronic obstructive pulmonary disease (COPD), yet the causal mechanisms and molecular mediators underlying this progression remain poorly understood. Multi-omics integration provides a powerful framework for prioritizing potential mediating proteins and disease-specific therapeutic candidates.

**Methods:**

This study integrated large-scale genetic and proteomic data using Mendelian randomization (MR) approaches to investigate the progression from EOA to COPD. Proteome-wide MR evaluated protein quantitative trait loci (pQTLs) in relation to EOA and COPD risk, with mediation analysis evaluating their roles and single-cell transcriptomics defining the cell-type-specific expression of the mediating proteins. Finally, colocalization, multi-tissue expression quantitative trait loci (eQTLs), and druggability assessment were used to prioritize potential disease-specific therapeutic targets.

**Results:**

Evidence from genetic instruments supports a causal relationship between EOA and COPD. Proteome-wide analyses of 7847 pQTLs identified 339 proteins with potential effects on EOA and 389 on COPD. Six proteins, KREMEN1, BLMH, CNTN5, IL1RN, MIA, and PILRA, showed statistically significant mediation effects in the EOA-to-COPD pathway. PILRA strongly colocalized at shared genetic loci between the two diseases and was significantly downregulated in macrophages from COPD patients. For disease-specific targets, immune-tissue eQTL validation supported ITPKA in EOA. Integration of druggability assessment with multi-tissue eQTL analyses prioritized FES, CCN3, NMI, and NMT1 as promising therapeutic candidates for COPD.

**Conclusion:**

These findings provide genetic evidence supporting a causal relationship between EOA and COPD, reveal putative mediating proteins, and prioritize therapeutic candidates with translational potential, offering new insights into pathogenesis, prevention, and intervention.

## Introduction

Chronic respiratory diseases (CRDs) constitute a major category of noncommunicable diseases and impose a substantial burden on global health and population well-being. Among CRDs, chronic obstructive pulmonary disease (COPD) accounts for the largest proportion of CRD-related mortality, whereas asthma exhibits the highest prevalence worldwide [1]. According to the Global Burden of Disease study [2], the age-standardized prevalence of asthma is 3340 cases per 100 000 population, with early-onset asthma (EOA), typically diagnosed during childhood or adolescence, accounting for a considerable proportion. In contrast, COPD predominantly affects older adults and has an age-standardized mortality of 45.22 per 100 000 [3]. Both conditions impact hundreds of millions of people globally and contribute substantially to disability-adjusted life years.

Beyond their substantial disease burden, asthma and COPD are epidemiologically associated. Longitudinal cohorts indicate that EOA is associated with both a markedly increased risk of subsequent COPD and an overall decline in lung function [4, 5], leading to more severe clinical outcomes. However, these observational findings are often confounded by various factors, limiting the ability to draw definitive conclusions regarding causality [6]. Prospective multi-omics cohort studies tracking the progression from EOA to adult COPD typically require long-term follow-up and substantial resources, which pose considerable challenges for large-scale implementation [7]. Therefore, the molecular mechanisms that facilitate the transition from asthma to COPD remain poorly characterized, hindering the development of effective early intervention strategies.

Therapeutically, current strategies mainly aim at symptom control and slowing disease progression [8, 9]. Although inhaled glucocorticoids and other conventional therapies can significantly relieve symptoms in some patients, a portion of severe cases respond poorly, and long-term or high-dose use may cause serious adverse effects, such as osteoporosis, thromboembolic events, and gastrointestinal perforation, which can markedly impact patients’ quality of life [10]. In recent years, molecular targeted therapies have provided new insights into disease mechanisms and enabled precision interventions. However, currently identified or approved targeted treatments remain limited [11, 12], mostly focusing on specific inflammatory pathways. For example, anti-immunoglobulin E (IgE) and anti-interleukin 5 therapies are effective only in a subset of severe asthma patients characterized by specific immunological profiles, such as high IgE or elevated blood eosinophils [11]. This highlights the urgent need to uncover novel molecular targets with broader and more reliable therapeutic potential.

Conventional epidemiological studies can identify potential drug targets, but many seemingly associated biomarkers are not truly causal, resulting in the failure of numerous drugs in phase II/III clinical trials due to insufficient efficacy [13]. Several studies [14, 15] have shown that genetically supported targets have a success rate at least twice as high as that of unsupported targets, with greater translational reliability, lower costs, and shorter development timelines, thereby significantly improving research efficiency. Building on these considerations, this study integrates extensive genetic and multi-omics data and employs a stepwise analytical approach centered on genetic causal inference, complemented by functional and expression-based evidence, to explore the potential causal relationship between EOA and COPD, suggest mediating proteins, and prioritize disease-specific therapeutic targets.

## Materials and methods

**Figure 1:**
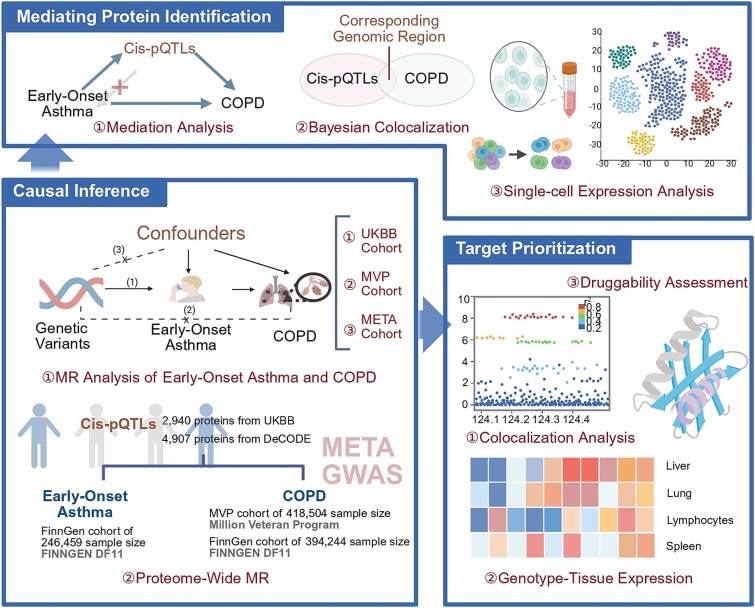
Overview of study design. A schematic representation of the study design, structured in three sequential stages: (i) evaluation of potential causal relationships: two-sample MR is applied to assess and validate the causal effect of EOA on COPD, while proteome-wide MR is conducted to identify *cis*-pQTLs associated with these diseases. (ii) Investigation of mediating proteins: candidate pQTLs are integrated into mediation analyses, with colocalization providing genetic validation of mediating loci. Single-cell RNA sequencing is leveraged to define cell-type-specific expression patterns of the mediating proteins. (iii) Prioritization of disease-specific therapeutic targets: Bayesian colocalization is used to confirm causal links between *cis*-pQTLs and disease, while multi-tissue eQTLs analyses combined with druggability assessment elucidate potential underlying biological mechanisms. (Created in BioRender.com. Yuhan, J. (2026) https://BioRender.com/44b7oia).

The study was structured in three stages. First, we evaluated potential causal relationships by applying two-sample Mendelian randomization (MR) to assess and validate the causal effect of EOA on COPD, and performing proteome-wide MR to identify *cis*-protein quantitative trait loci (pQTLs) associated with these diseases. Second, we investigated mediating proteins by incorporating pQTLs into mediation analyses, validating mediating loci through colocalization, and delineating the cell-type-specific expression patterns using single-cell RNA sequencing (scRNA-seq). Third, we prioritized disease-specific therapeutic targets by confirming causal links between pQTLs and disease via Bayesian colocalization, multi-tissue *cis*-expression quantitative trait loci (eQTLs) analyses, and assessing druggability to shed light on potential biological mechanisms. The overall study design is illustrated in Fig. 1.

### Integration and meta-analysis of genome-wide association studies

All genome-wide association study (GWAS) datasets were derived from large-scale cohort studies, with EOA data sourced from the FinnGen [16], comprising 7537 cases and 238 922 controls. GWAS data for COPD were sourced from three cohorts: the Million Veteran Program (MVP; 103 054 cases and 315 450 controls) [17], FinnGen (21 617 cases and 372 627 controls) [16], and the UK Biobank (UKBB; 11 536 cases and 408 995 controls) [18]. pQTLs were obtained from two large-scale sources: the UKBB and deCODE Genetics. The UKBB dataset, generated by the Pharma Proteomics Project [19], quantified 2940 plasma proteins in 34 557 participants using the Olink platform, while the deCODE dataset included 35 559 Icelandic individuals [20] with 4907 aptamers measured via the SomaScan platform. All participants across cohorts were of European ancestry. Among these datasets, 1815 proteins were measured in both cohorts, providing an opportunity for cross-cohort validation. Further details are provided in Table S1.

To increase statistical power, we performed a meta-analysis of COPD GWAS data using METAL [21] under a fixed-effects model weighted by effective sample size. Heterogeneity across cohorts was assessed using Cochran’s Q statistic and I^2^. To ensure the reliability of the results, genomic control was applied to correct for population stratification and other confounding factors. Linkage disequilibrium score regression (LDSC) [22] was subsequently performed to evaluate the overall quality of the GWAS results and to distinguish inflation driven by true polygenic signals from residual nonbiological confounding.

Notably, to avoid sample overlap with the exposure GWAS, two separate meta-analyses for COPD were conducted: META1 Cohort, combining the UKBB and MVP cohorts to assess the association with EOA; and META2 Cohort, combining the FinnGen and MVP cohorts for the proteome-wide MR analyses.

### Two-sample Mendelian randomization

Two-sample MR analyses were conducted using the “TwoSampleMR” R package, with the study design adhering to the three core assumptions of MR: relevance, independence, and exclusion restriction [23]. Instrumental variables (IVs) were selected using strict criteria: (i) single-nucleotide polymorphisms (SNPs) significantly associated with exposure (*P* ≤ 5 × 10^−8^) and not significantly associated with outcome (*P* > 5 × 10^−8^); (ii) linkage disequilibrium clumping was performed using UK10K [24], retaining independent SNPs with r^2^ < 0.001 for each exposure. Notably, to minimize potential pleiotropic effects arising from the shared genetic architecture between EOA and COPD, we used FUMA [25] to annotate the IVs for exposures and excluded overlapping SNPs. For the proteome-wide MR analysis, *cis*-pQTLs were selected as IVs to reduce pleiotropy and improve biological interpretability. *Cis*-pQTLs were defined as SNPs located within a 1 Mb window flanking the transcription start site of the corresponding protein-coding gene. Subsequently, MR-PRESSO [26] was used to assess pleiotropy and remove outlier IVs. IVs with F-statistics <10 were considered weak instruments and excluded from further analyses.

Multiple analytical methods and sensitivity analyses were applied to ensure robustness. For the primary MR analysis, the inverse variance weighted (IVW) method [27] was employed for proteins with multiple IVs, while the Wald ratio method [28] was applied for proteins with a single IV. Subsequently, sensitivity analyses were performed to assess potential heterogeneity and horizontal pleiotropy. In cases where horizontal pleiotropy was detected, MR-Egger [29] was employed to evaluate potential bias. When significant heterogeneity was observed, multiplicative random-effects IVW [27] and weighted median [30] methods were applied to ensure the robustness of the results. Furthermore, the complementary methods, such as the simple mode and weighted mode [31], were also considered to confirm the findings. To reduce the risk of type I error, the false discovery rate (FDR) was applied to correct for multiple testing [32]. These strategies collectively ensure that the results are not influenced by specific methodological choices or weak instruments.

### Proteome-wide mediation analyses and colocalization analyses

This study conducted mediation analyses within the two-sample MR framework. This approach requires the following assumptions to be met [23, 33]: (i) a significant association exists between EOA and COPD; (ii) the mediating protein is independently associated with COPD, indicating a direct effect; and (iii) the EOA is associated with the mediating protein, without evidence of reverse causation. To minimize bias and reduce the risk of inflated false-positive findings due to sample overlap [34], GWAS sources were systematically integrated to ensure cohort independence across exposure, mediating protein, and outcome datasets. Finally, confidence intervals for the mediation proportion were computed using the Delta method.

Bayesian colocalization was performed using the R package “coloc” [35] to assess whether phenotypes share a common causal variant. This approach estimates the posterior probabilities for five mutually exclusive hypotheses. Particular emphasis was placed on the posterior probability of hypothesis 4 (PP.H4), which quantifies the probability that both traits share the same causal variant. A PP.H4 ≥ 0.7 was considered indicative of strong evidence for colocalization.

### Single-cell type differential expression analyses

To validate the expression of mediating proteins in bronchoalveolar lavage fluid (BALF) cells and their cell type-specific patterns, scRNA-seq data (GSE171541) [36] were analyzed. This dataset includes BALF samples from three COPD patients and six healthy controls. Raw data preprocessing was performed using the R package “Seurat” [37], which included quality filtering, normalization, log transformation, and batch correction. Dimensionality reduction and visualization were conducted via principal component analysis and uniform manifold approximation and projection (UMAP), enabling identification of cellular heterogeneity. Clusters were biologically annotated based on established cell-type-specific marker genes. Subsequently, genes encoding the significant mediating proteins were extracted, and their expression profiles were characterized across distinct cell types. Differential expression analysis comparing COPD and control groups was conducted within each cell type using the Wilcoxon rank-sum test. Genes exhibiting an absolute log₂ fold change greater than .25 and an FDR-adjusted *P* below .05 were deemed differentially expressed.

### Druggability evaluation and cross-tissue gene expression validation

To prioritize potential therapeutic targets, related proteins for EOA and COPD were evaluated separately. Proteins that remained significant after FDR correction in the proteome-wide MR analyses and exhibited a colocalization probability ≥.7 were considered high-confidence candidates. To assess druggability, we retrieved information on associated drugs, approval status, therapeutic indications, and mechanisms of action from DrugBank [38]. For proteins lacking known drug targets, 3D structures were predicted using AlphaFold [39] to facilitate identification of potential ligand-binding sites: binding pockets of enzymes were evaluated using DoGSiteScorer [40], whereas hotspot regions of non-enzymes were assessed using FTMap [41]. In addition, protein–protein interactions (PPIs) were analyzed using STRING [42] to provide functional context and explore potential opportunities for indirect targeting.

To validate the tissue-specific genetic regulation of high-confidence proteins and further evaluate their potential as therapeutic targets, we analyzed tissue-specific *cis*-eQTLs from the GTEx v10 project [43]. Protein-coding *cis*-eQTLs from COPD and asthma-relevant tissues, including lung, systemic tissues (such as peripheral blood and liver), and immune tissues (such as spleen and lymphocytes), were selected for MR analyses. Additionally, to ensure robust instrument selection and maximize the representation of protein-coding genes as valid IVs within each tissue [44], *cis*-eQTLs were filtered using a liberal significance threshold (*P* ≤ 5 × 10^−5^) combined with an instrument strength criterion (F-statistic >10).

## Results

### Putative causal relationships between early-onset asthma and COPD

After excluding nine SNPs with potential shared genetic architecture between EOA and COPD, as detailed in Table S2, causal relationships were investigated across three datasets: the discovery cohort (MVP), the validation cohort (UKBB), and the META1 cohort, which represents a meta-analysis of the MVP and UKBB GWAS. Specifically, the META1 analysis included 839 035 individuals and evaluated 28 290 080 common genetic variants under a fixed-effects model. A total of 599 433 variants showing significant heterogeneity were excluded. After applying genomic control, the GWAS results showed a genomic inflation factor of 1.307 and a mean χ^2^ of 1.469. LDSC yielded an intercept of 0.867 (standard error 0.009) with a ratio <0, indicating that the observed inflation was primarily driven by true polygenic signals rather than systematic biases. These metrics are consistent with those reported in large-scale GWAS meta-analyses of complex traits [45], reflecting the polygenic and complex genetic architecture of COPD. Additionally, as MR analyses rely on independent genome-wide significant variants and ratio-based estimation, they are relatively robust to inflation arising from polygenicity [22].

**Figure 2:**
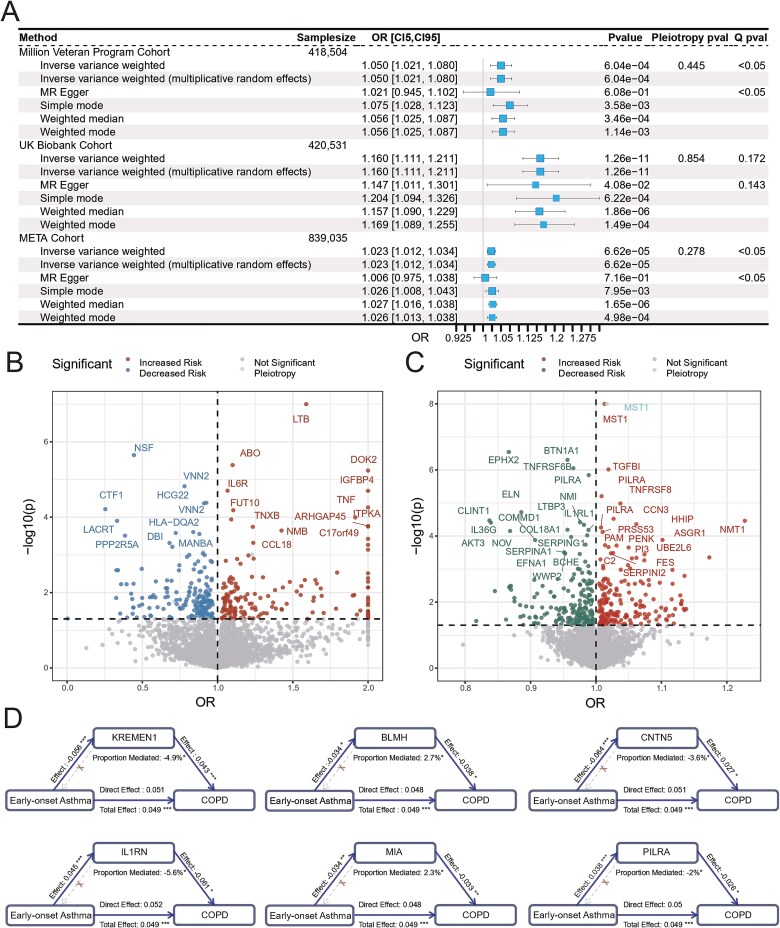
Summary results of two-sample MR and proteome-wide association studies. (A) Two-sample MR results from EOA and COPD. Analyses were performed using the discovery cohort (MVP), the validation cohort (UKBB), and the META1 cohort (a meta-analysis of MVP and UKBB) to assess and validate causal effects. The central forest plot displays the estimated effect size of EOA on COPD, with squares representing ORs and horizontal lines indicating 95% confidence intervals. (B) Proteome-wide MR analyses for EOA. The *Y*-axis represents the negative base-10 logarithm of the *P*-value, and the *X*-axis shows OR. Points are color-coded by association direction (OR > 1 or OR < 1) and pleiotropy levels. Labeled points indicate proteins with statistically significant associations after FDR correction (FDR-adjusted *P* < .05). (C) Proteome-wide MR analyses for COPD. The *Y*-axis shows the negative base-10 logarithm of the *P*-value, and the *X*-axis shows OR. Points are color-coded by association direction (OR > 1 or OR < 1) and pleiotropy levels. Labeled points indicate proteins with statistically significant associations after FDR correction (FDR-adjusted *P* < .05). (D) Mediation effects of circulating proteins linking EOA to COPD. Each pathway is annotated with its corresponding effect size. Significance levels are denoted as follows: ^*^(*P* < .05), ^**^ (*P* < .01), and ^***^ (*P* < .001).

Subsequently, in the discovery cohort, the IVW analysis indicated that EOA was a significant risk factor for COPD (odds ratio, OR = 1.050, 95% CI: 1.021–1.080, *P* = 6.04 × 10^−4^). Sensitivity analyses suggested that horizontal pleiotropy was well controlled. Although mild heterogeneity was detected, the causal estimates remained statistically significant after correction using both the multiplicative random-effects IVW and weighted median approaches. Furthermore, multiple MR approaches identified significant associations in a concordant direction. These findings were replicated in the validation cohort and the META1 cohort, reinforcing their robustness. Collectively, the results support EOA as a risk factor for COPD and suggest a potential causal relationship between the two conditions. Detailed results are provided in Fig. 2A.

### Proteome-wide analyses of early-onset asthma and COPD

In parallel, proteome-wide MR was conducted to evaluate potential causal relationships of 7847 plasma proteins with EOA and COPD. For EOA, after selecting *cis*-pQTLs as IVs and excluding proteins without valid SNPs, a final analysis was performed on 3813 proteins. Among the analyzed proteins, 339 exhibited statistically significant causal effects. After controlling for multiple testing using the FDR correction, 23 proteins retained significance, including lymphotoxin-beta (LTB), interleukin-6 receptor subunit alpha (IL6R), and C-C motif chemokine 18 (CCL18), as illustrated in Fig. 2B. Notably, pantetheine hydrolase Vanin 2 (VNN2) exhibited consistent and significant effects across both protein cohorts, highlighting its potential relevance to EOA pathogenesis.

For COPD, the META2 cohort was used to ensure adequate statistical power while avoiding sample overlap with the datasets of pQTLs. The analysis integrated data from 812 748 individuals, encompassing 27 931 600 common genetic variants, of which 529 317 showed significant heterogeneity and were excluded. After genomic control, the GWAS showed a genomic inflation factor of 1.355 (mean χ^2^ = 1.525); the LDSC intercept was 0.880 (standard error 0.009), with a ratio <0. *Cis*-pQTLs were selected as IVs, yielding a final analytical set of 3782 proteins. In total, 389 proteins demonstrated statistically significant effects on COPD, of which 36 remained significant after FDR correction and passed sensitivity analyses, including elastin (ELN), alpha-1-antitrypsin (SERPINA1), and elafin (PI3), as shown in Fig. 2C. Notably, paired immunoglobulin-like type 2 receptor alpha (PILRA) and CCN family member 3 (CCN3, also known as NOV) exhibited consistent and significant effects after FDR correction in both independent protein cohorts, further highlighting their robustness and relevance to COPD pathogenesis. To ensure full transparency, all results from the proteome-wide analyses of EOA and COPD have been made publicly available and can be downloaded from Figshare (https://doi.org/10.6084/m9.figshare.30983989).

### Mediation analyses and cell-type-specific expression

**Figure 3:**
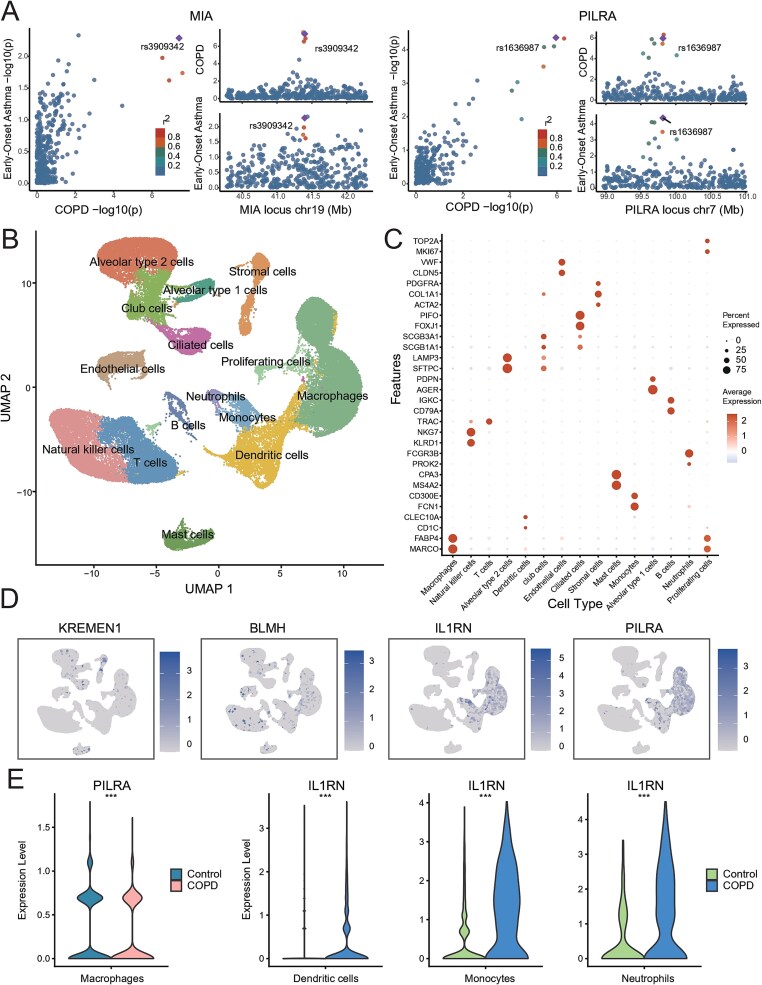
Downstream analyses of mediating proteins. (A) Colocalization results for the MIA and PILRA gene regions in EOA and COPD. Left panel: *X*-axis shows the negative base-10 logarithm of the *P*-value for COPD-associated gene regions, *Y*-axis shows the negative base-10 logarithm of the *P*-value for EOA-associated gene regions. Right panel: *X*-axis represents genomic positions; *Y*-axis shows the negative base-10 logarithm of the *P*-value. Point colors indicate the strength of the correlation between association signals. (B) Clustering of single cells using UMAP identified 15 distinct cell types. (C) Expression profiles of typical marker genes across the identified cell types. (D) Expression of mediating protein-encoding genes within the UMAP clusters, with color intensity representing expression levels. (E) Differential expression analyses of candidate protein-coding genes between COPD patients and healthy controls within cell-type-specific enriched populations. Statistical significance is assessed using Wilcoxon rank-sum tests. Significance levels after FDR correction are indicated by ^*^ (*P* < .05), ^**^ (*P* < .01), and ^***^ (*P* < .001).

To further investigate the mechanisms by which EOA contributes to COPD pathogenesis, mediation analyses were conducted within the MR framework. A total of 78 candidate mediating proteins were identified, among which 6 proteins: kremen protein 1 (KREMEN1), bleomycin hydrolase (BLMH), contactin-5 (CNTN5), interleukin-1 receptor antagonist protein (IL1RN), melanoma-derived growth regulatory protein (MIA), and PILRA, exhibited statistically significant mediation effects. The corresponding results are shown in Fig. 2D and Table S3. To account for the potential of false positives due to weak mediation effects, colocalization was performed to validate the genetic associations of EOA and COPD within the gene regions encoding these proteins. Our results revealed that MIA (PP.H4 = 0.68) and PILRA (PP.H4 = 0.94) provided compelling evidence of colocalization, suggesting a shared genetic pathway. Detailed results are provided in Fig. 3A and Table S4.

We further investigated the potential mechanisms of the mediating proteins by examining their cell-type-specific expression profiles in BALF obtained from COPD patients. The delineation of specific cellular clusters along with their respective marker genes is presented in Fig. 3B and C. It was observed that KREMEN1, BLMH, IL1RN, and PILRA were all expressed in BALF, as illustrated in Fig. 3D. Notably, when comparing COPD patients to healthy controls, PILRA expression was significantly downregulated in macrophages, whereas IL1RN showed a trend toward upregulation in dendritic cells, monocytes, and neutrophils. These findings suggest that PILRA and IL1RN may play differential roles in disease-associated immune regulation. Comprehensive results are shown in Fig. 3E with additional details available in Table S5.

### Assessment of potential therapeutic targets for early-onset asthma

**Figure 4:**
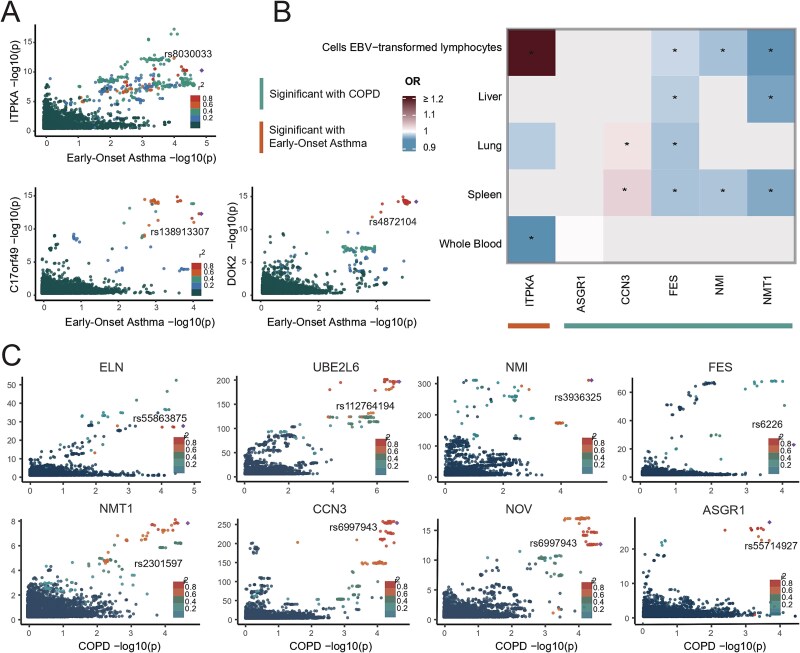
Potential disease-specific drug targets for EOA and COPD. (A) Colocalization analysis of high-confidence proteins for EOA. The *X*-axis represents the negative base-10 logarithm of the *P*-value for EOA-associated variants, while the *Y*-axis shows the negative base-10 logarithm of the *P*-value for corresponding *cis*-pQTLs. Point colors indicate the strength of correlation between *cis*-pQTLs and EOA association signals. (B) Heatmap illustrating the results for high-confidence protein-coding genes across tissue-specific transcriptomes for EOA and COPD. Colors indicate ORs, and asterisks denote statistically significant associations after FDR correction (*P* < .05). (C) Colocalization analysis of high-confidence proteins for COPD. The *X*-axis represents the negative base-10 logarithm of the *P*-value for COPD-associated variants, while the *Y*-axis shows the negative base-10 logarithm of the *P*-value for corresponding pQTLs. Point colors indicate the strength of correlation between *cis*-pQTLs and COPD association signals.

To assess whether the genetic *cis*-pQTLs and disease-associated loci reflected the same causal variant, colocalization analyses were performed on the 23 proteins that remained significant after FDR correction. Three proteins exhibited strong colocalization evidence (PP.H4 ≥ 0.7), including inositol-trisphosphate 3-kinase A (ITPKA), BPTF-associated chromatin complex component 1 (C17orf49), and docking protein 2 (DOK2), as shown in Fig. 4A and Table S6.

To further validate the regulatory effects of candidate targets in disease-relevant tissues, MR analyses were conducted using *cis*-eQTLs of the encoding genes. ITPKA showed statistically significant causal associations in whole blood and Epstein-Barr virus (EBV)-transformed lymphocytes, supporting its potential role in disease pathogenesis. Detailed results are provided in Fig. 4B and Table S7. Druggability assessment revealed that ITPKA, as an enzyme, possesses an ATP-like binding site, which is crucial for its enzymatic activity. Existing research on kinase inhibitors further supports its potential druggability, as outlined in Table S8.

### Assessment of potential therapeutic targets for COPD

For COPD, colocalization was conducted on the 36 proteins that remained significant after FDR correction, identifying 7 unique proteins with strong colocalization evidence, including CCN3 (also known as NOV), glycylpeptide N-tetradecanoyltransferase 1 (NMT1), ubiquitin/ISG15-conjugating enzyme E2 L6 (UBE2L6), Asialoglycoprotein receptor 1 (ASGR1), N-myc-interactor (NMI), and ELN. Notably, CCN3 demonstrated colocalization in both the UKBB and deCODE cohorts, reinforcing its candidacy as a key protein in COPD pathogenesis, as shown in Fig. 4C and Table S6.

Furthermore, multi-tissue eQTL analyses revealed statistically significant causal associations between the *cis*-eQTLs of FES and CCN3 in lung and immune tissues and COPD. Additionally, *cis*-eQTLs of NMI and NMT1 in immune tissues also demonstrated significant relationships with the disease. These findings, which reinforce the potential role of these proteins, are detailed in Fig. 4B and Table S7.

Druggability assessment indicated that several candidate proteins have previously been investigated as therapeutic targets in other diseases, offering potential opportunities for drug repurposing and the identification of new targets for COPD. For example, the FES inhibitor Lorlatinib is widely used in the treatment of certain types of lung cancer. The NMT1 inhibitor Zelenirstat has demonstrated significant efficacy and promising therapeutic potential across multiple solid tumors, as shown in Table S8.

In contrast, no approved drugs currently target CCN3. However, structural pocket prediction identified 10 potential binding sites, 4 of which showed high composite scores (drug score > 0.7) across volume, surface, and depth, suggesting potential druggability, as shown in Table S9 and Fig. S1. For NMI, FTMap predicted binding hotspots primarily at residues A58 and A253. The A58 region contributed most strongly to nonbonded interactions, while A253 and adjacent residues were enriched in hydrogen bonds, suggesting potential functional binding interfaces, as illustrated in Figs S2–S4. Additionally, the PPI network revealed 10 high-confidence interacting partners, as shown in Fig. S5.

## Discussion

This study integrated large-scale multi-omics data within a multistage genetic inference approach to systematically investigate the relationship between EOA and COPD. The analyses yielded consistent genetic evidence indicating EOA as a causal risk factor for COPD. Proteome-wide and mediation analyses further identified several plasma proteins, including MIA, IL1RN, and PILRA, which are consistent with statistically supported candidates as potential mediating proteins in the pathway linking EOA to COPD. Integration of tissue-specific expression analyses and druggability assessment highlighted ITPKA in EOA and FES, CCN3, NMI, and NMT1 in COPD as prioritized candidates for further experimental investigation. These results, based on data-driven and statistical analyses, offer insights into disease pathogenesis and provide genetically informed evidence that may support future mechanistic and therapeutic investigations.

The causal relationship between EOA and COPD has long been hypothesized. However, observational studies are often confounded by factors [46] such as smoking, occupational exposures, and environmental pollution, making it difficult to establish causality. As early as 1961, the Dutch hypothesis [47] suggested that asthma and COPD may represent different manifestations of a shared spectrum of chronic airway disease with overlapping genetic susceptibility. Clinically, asthma and COPD can coexist, forming asthma-COPD overlap syndrome, a condition generally associated with poorer clinical outcomes, although no unified international definition or diagnostic criteria have been established [48]. In this context, our findings provide genetic evidence that EOA may contribute causally to the development of COPD and even potentially to asthma-COPD overlap syndrome, thereby reinforcing the concept of a shared disease spectrum. These results highlight the importance of early airway intervention in childhood, not only for optimizing asthma control but also for reducing the risk of long-term chronic respiratory morbidity, offering new perspectives for precision prevention and management strategies.

Furthermore, this study applied MR to identify six potential plasma proteins associated with EOA and COPD. Compared with other causal inference frameworks, such as multivariable regression or structural equation modeling, two-step MR enables the simultaneous causal assessment of thousands of proteins in large population-based samples [49] and relies less on explicit modeling of unmeasured confounding and temporal ordering [50], thereby providing a clearer inference of the causal pathway linking EOA, plasma proteins, and COPD. Nevertheless, the inference remains substantially influenced by the choice of IVs and potential pleiotropy. Specifically, IVs for KREMEN1 (I^2^ = 19.41) and MIA (I^2^ = 13.52) exhibited low heterogeneity, while the remaining proteins showed no significant heterogeneity, supporting overall stability. MR-Egger intercepts for IL1RN, MIA, PILRA, CNTN5, and KREMEN1 were nonsignificant, and these instruments were mainly functionally well-characterized *cis*-pQTLs, suggesting a low likelihood of directional pleiotropy. In contrast, BLMH showed a borderline intercept (*P* = .049); although its causal estimate aligned with the primary analysis, residual pleiotropy cannot be fully excluded, warranting cautious interpretation.

Additionally, several proteins, including PILRA, IL1RN, and MIA, were expressed in COPD BALF, suggesting a potential role in disease processes. Of particular interest, single-cell expression analysis reveals that PILRA is significantly downregulated in macrophages from COPD patients. In asthma, macrophages tend to polarize toward the M2 phenotype [51], exhibiting enhanced regulatory activity and Th2-dominant inflammation, whereas in COPD, macrophages are increased and polarized toward the M1 phenotype [52, 53], characterized by persistent inflammation, impaired phagocytic and clearance functions, and elevated oxidative stress. PILRA, an inhibitory receptor containing an immunoreceptor tyrosine-based inhibitory motif [54], can recruit SHP-1 and SHP-2 upon ligand binding to suppress immune cell activation and maintain immune homeostasis. Taken together, we speculate that PILRA may play a role in macrophage polarization and inflammatory regulation in the context of EOA and COPD, which warrants further mechanistic investigation.

Although EOA and COPD share certain genetic and pathological pathways, substantial evidence indicates significant heterogeneity between the two conditions [55], including differences in clinical manifestations, inflammatory profiles, and molecular mechanisms, which ultimately determine disease-specific therapeutic targets. In the present study, proteome-wide analyses identified several proteins with putative causal effects on EOA, many of which are supported by existing evidence. For example, animal experiments demonstrate that blocking IL6R with monoclonal antibodies alleviates allergen-induced airway inflammation and hyperresponsiveness [56], and CCL18 is associated with eosinophilic inflammation and severe asthma phenotypes [57]. Furthermore, ITPKA was validated in our immune-related tissue eQTLs analyses, highlighting its potential involvement in airway disease pathways. Previous genetic studies have linked ITPKA to allergic diseases [58], including asthma, allergic rhinitis, and atopic dermatitis. Functionally, ITPKA is a key regulator of intracellular calcium signaling and the actin cytoskeleton, and its dysregulation may contribute to airway hyperresponsiveness and chronic inflammation in asthma [59]. Structurally, the well-defined ATP-like binding pocket of ITPKA renders it potentially druggable.

For COPD, proteome-wide analyses have identified 36 associated proteins, including ELN, SERPINA1, and PI3, which play pivotal roles in disease pathology. PI3, as a specific inhibitor of neutrophil and pancreatic elastases [60], reduces tissue damage, fibrosis, and structural remodeling. SERPINA1 deficiency further accelerates elastin degradation, exacerbating emphysematous changes [61]. Further eQTL analyses validated FES, CCN3, NMI, and NMT1 in lung tissues and various immune-related tissues. Specifically, FES, a tyrosine kinase, participates in inflammation-related signaling pathways, such as PI3K-Akt and JAK-STAT, suggesting a potential role in chronic airway inflammation and tissue remodeling [62]. Its targeted drug, fostamatinib, has demonstrated efficacy in modulating inflammation and alleviating acute respiratory distress syndrome in severe COVID-19 cases. NMT1 is involved in viral replication and immune signaling, and its targeted drug Zelenirstat is the first drug for this specific target, used in lung cancer treatment, supporting its candidacy for further pharmacological evaluation and potential drug repurposing research. Among proteins lacking current therapeutic agents, CCN3 is notable. In mouse models, CCN3 deficiency in pulmonary endothelial cells impairs angiogenesis and promotes fibrosis [63], whereas recombinant CCN3 restores vascular function and reduces fibrosis [64]. Structural predictions reveal several high-confidence binding pockets, suggesting its potential as a small-molecule drug target. Furthermore, NMI plays a crucial role in inflammation signaling pathways and is involved in immune response and apoptosis during viral infections [65], contributing to COPD progression. PPI results and related studies indicate [66] that NMI interacts with multiple transcription factors to regulate immune responses and inflammatory pathways, suggesting that NMI may be indirectly targetable through allosteric modulation or protein complex-mediated mechanisms.

### Strengths and limitations

To our knowledge, this study represents the largest integrative proteomic analysis to date on EOA and COPD, with several notable advantages. Methodologically, unlike conventional MR-pQTL studies that primarily evaluate individual protein-disease associations [67], our study investigates the specific biological question of whether EOA contributes to COPD development via intermediate molecular mechanisms. We implemented a stepwise causal inference framework that transforms single-step causal inference into a directional, mechanistically informative chain. From an evidentiary perspective, MR analyses are inherently limited by factors such as potential pleiotropy, highlighting the need for multidimensional validation [68]. Previous studies [14] have shown that stronger causal evidence improves the likelihood of drug target success. To address these challenges, we applied multilayer complementary validation across several methods and databases, thereby reducing potential bias and enhancing robustness. Biologically and translationally, large-scale proteogenomic data not only facilitate systematic prioritization of candidate targets but also guide avoiding inefficient drug development when causal effects of certain biomarkers are excluded by genetic evidence [13]. Therefore, all available results, both positive and negative, may serve as valuable references for future studies.

However, several limitations should be acknowledged. First, MR analyses rely primarily on GWAS data. Although meta-analyses of COPD GWAS were conducted to enhance the statistical power of SNP associations, the limited availability of genetic variant data for exposures remains an unavoidable challenge. Second, mediation MR relies on assumptions such as linearity and the absence of exposure–mediator interaction, which may not fully hold in complex biological and environmental systems [69]. The resulting mediation proportion should be interpreted as a statistical estimate rather than a direct measure of biological effect. In addition, to minimize population stratification bias, the study sample was restricted to individuals of European ancestry, limiting the generalizability of the findings to other racial or ethnic populations. Furthermore, due to the limitations of available GWAS cohorts and to avoid sample overlap, the mediation results have not yet been validated in independent datasets. Finally, asthma and COPD are highly heterogeneous diseases, with different subtypes and endotypes potentially exhibiting distinct pathological and molecular mechanisms [70]. The present study was therefore limited in its ability to explore subtype-specific mechanisms.

It is important to emphasize that the proteins highlighted in this study were prioritized based on statistical genetic evidence and computational predictions, reflecting their potential involvement in genetic causal pathways, tissue-specific regulation, and structural features indicative of druggability. Future studies could integrate functional experiments and independent cohort analyses to validate and quantify the framework’s incremental value, and to assess its translational potential at the empirical level.

**Key Points**

- Our study provides large-scale genetic evidence supporting a potential causal association between EOA and COPD.
- Integrative multi-omics analyses highlight several plasma proteins (KREMEN1, BLMH, CNTN5, IL1RN, MIA, and PILRA) that may mediate the EOA-COPD pathway, with PILRA further supported by colocalization.
- Proteomic analyses prioritize therapeutic candidates for EOA (ITPKA) and COPD (FES, CCN3, NMI, and NMT1).

## Supplementary Material

Supplemental_Figures_bbag209

Supplemental_Tables_bbag209

## Data Availability

The proteome-wide analyses of EOA and COPD have been made publicly available and can be accessed for download at Figshare (https://doi.org/10.6084/m9.figshare.30983989), while the remaining results are provided in the Supplementary Tables.

## References

[1] GBD 2019 Chronic Respiratory Diseases Collaborators . Global burden of chronic respiratory diseases and risk factors, 1990-2019: an update from the global burden of disease study 2019. *EClinicalMedicine* 2023;59:101936. 10.1016/j.eclinm.2023.101936 37229504 PMC7614570

[2] Yuan L, Tao J, Wang J et al. Global, regional, national burden of asthma from 1990 to 2021, with projections of incidence to 2050: a systematic analysis of the global burden of disease study 2021. *EClinicalMedicine* 2025;80:103051. 10.1016/j.eclinm.2024.103051 39867965 PMC11764843

[3] Wang Z, Lin J, Liang L et al. Global, regional, and national burden of chronic obstructive pulmonary disease and its attributable risk factors from 1990 to 2021: an analysis for the global burden of disease study 2021. *Respir Res* 2025;26:2. 10.1186/s12931-024-03051-2 39748260 PMC11697803

[4] McGeachie MJ, Yates KP, Zhou X et al. Patterns of growth and decline in lung function in persistent childhood asthma. *N Engl J Med* 2016;374:1842–52. 10.1056/NEJMoa1513737 27168434 PMC5032024

[5] Tai A, Tran H, Roberts M et al. The association between childhood asthma and adult chronic obstructive pulmonary disease. *Thorax* 2014;69:805–10. 10.1136/thoraxjnl-2013-204815 24646659

[6] Grimes DA, Schulz KF. Bias and causal associations in observational research. *Lancet* 2002;359:248–52. 10.1016/S0140-6736(02)07451-2 11812579

[7] Kayaba K . Overcoming the difficulties of cohort studies. *J Epidemiol* 2013;23:156–7. 10.2188/jea.JE20120225 23524526 PMC3700261

[8] Venkatesan P . 2025 GINA report for asthma. *Lancet Respir Med* 2025;13:e41–2. 10.1016/S2213-2600(25)00242-5 40582369

[9] Singh D, Stockley R, Anzueto A et al. GOLD science committee recommendations for the use of pre- and post-bronchodilator spirometry for the diagnosis of COPD. *Eur Respir J* 2025;65:2401603. 10.1183/13993003.01603-2024 39638416 PMC11799884

[10] Pandya D, Puttanna A, Balagopal V. Systemic effects of inhaled corticosteroids: an overview. *Open Respir Medi J* 2014;8:59–65. 10.2174/1874306401408010059 PMC4319197 25674175

[11] Pfeffer PE, Ali N, Murray R et al. Comparative effectiveness of anti-IL5 and anti-IgE biologic classes in patients with severe asthma eligible for both. *Allergy* 2023;78:1934–48. 10.1111/all.15711 36929509

[12] Pongdee T, Li JT. Omalizumab safety concerns. *J Allergy Clin Immunol* 2025;155:31–5. 10.1016/j.jaci.2024.11.005 39542143

[13] Mokry LE, Ahmad O, Forgetta V et al. Mendelian randomisation applied to drug development in cardiovascular disease: a review. *J Med Genet* 2015;52:71–9. 10.1136/jmedgenet-2014-102438 25515070

[14] Minikel EV, Painter JL, Dong CC et al. Refining the impact of genetic evidence on clinical success. *Nature* 2024;629:624–9. 10.1038/s41586-024-07316-0 38632401 PMC11096124

[15] Nelson MR, Tipney H, Painter JL et al. The support of human genetic evidence for approved drug indications. *Nat Genet* 2015;47:856–60. 10.1038/ng.3314 26121088

[16] Kurki MI, Karjalainen J, Palta P et al. FinnGen provides genetic insights from a well-phenotyped isolated population. *Nature* 2023;613:508–18. 10.1038/s41586-022-05473-8 36653562 PMC9849126

[17] Verma A, Huffman JE, Rodriguez A et al. Diversity and scale: genetic architecture of 2068 traits in the VA million veteran program. *Science* 2024;385:eadj1182. 10.1126/science.adj1182 39024449 PMC12857194

[18] Bycroft C, Freeman C, Petkova D et al. The UK biobank resource with deep phenotyping and genomic data. *Nature* 2018;562:203–9. 10.1038/s41586-018-0579-z 30305743 PMC6786975

[19] Sun BB, Chiou J, Traylor M et al. Plasma proteomic associations with genetics and health in the UK biobank. *Nature* 2023;622:329–38. 10.1038/s41586-023-06592-6 37794186 PMC10567551

[20] Eldjarn GH, Ferkingstad E, Lund SH et al. Large-scale plasma proteomics comparisons through genetics and disease associations. *Nature* 2023;622:348–58. 10.1038/s41586-023-06563-x 37794188 PMC10567571

[21] Willer CJ, Li Y, Abecasis GR. METAL: fast and efficient meta-analysis of genomewide association scans. *Bioinformatics* 2010;26:2190–1. 10.1093/bioinformatics/btq340 20616382 PMC2922887

[22] Bulik-Sullivan BK, Loh PR, Finucane HK et al. LD score regression distinguishes confounding from polygenicity in genome-wide association studies. *Nat Genet* 2015;47:291–5. 10.1038/ng.3211 25642630 PMC4495769

[23] de Leeuw C, Savage J, Bucur IG et al. Understanding the assumptions underlying Mendelian randomization. *Eur J Hum Genet* 2022;30:653–60. 10.1038/s41431-022-01038-5 35082398 PMC9177700

[24] UK10K Consortium, Walter K, Min JL et al. The UK10K project identifies rare variants in health and disease. *Nature* 2015;526:82–90. 10.1038/nature14962 26367797 PMC4773891

[25] Watanabe K, Taskesen E, van Bochoven A et al. Functional mapping and annotation of genetic associations with FUMA. *Nat Commun* 2017;8:1826. 10.1038/s41467-017-01261-5 29184056 PMC5705698

[26] Verbanck M, Chen CY, Neale B et al. Detection of widespread horizontal pleiotropy in causal relationships inferred from Mendelian randomization between complex traits and diseases. *Nat Genet* 2018;50:693–8. 10.1038/s41588-018-0099-7 29686387 PMC6083837

[27] Verduijn M, Siegerink B, Jager KJ et al. Mendelian randomization: use of genetics to enable causal inference in observational studies. *Nephrol Dial Transplant* 2010;25:1394–8. 10.1093/ndt/gfq098 20190244

[28] Harbord RM, Didelez V, Palmer TM et al. Severity of bias of a simple estimator of the causal odds ratio in Mendelian randomization studies. *Stat Med* 2013;32:1246–58. 10.1002/sim.5659 23080538

[29] Burgess S, Thompson SG. Interpreting findings from Mendelian randomization using the MR-Egger method. *Eur J Epidemiol* 2017;32:377–89. 10.1007/s10654-017-0255-x 28527048 PMC5506233

[30] Bowden J, Davey Smith G, Haycock PC et al. Consistent estimation in Mendelian randomization with some invalid instruments using a weighted median estimator. *Genet Epidemiol* 2016;40:304–14. 10.1002/gepi.21965 27061298 PMC4849733

[31] Hartwig FP, Davey Smith G, Bowden J. Robust inference in summary data Mendelian randomization via the zero modal pleiotropy assumption. *Int J Epidemiol* 2017;46:1985–98. 10.1093/ije/dyx102 29040600 PMC5837715

[32] Benjamini Y, Hochberg Y. Controlling the false discovery rate: a practical and powerful approach to multiple testing. *J R Stat Soc B Methodol* 1995;57:289–300. 10.1111/j.2517-6161.1995.tb02031.x

[33] Ye CJ, Liu D, Chen ML et al. Mendelian randomization evidence for the causal effect of mental well-being on healthy aging. *Nat Hum Behav* 2024;8:1798–809. 10.1038/s41562-024-01905-9 38886532

[34] Burgess S, Davies NM, Thompson SG. Bias due to participant overlap in two-sample Mendelian randomization. *Genet Epidemiol* 2016;40:597–608. 10.1002/gepi.21998 27625185 PMC5082560

[35] Giambartolomei C, Vukcevic D, Schadt EE et al. Bayesian test for colocalisation between pairs of genetic association studies using summary statistics. *PLoS Genet* 2014;10:e1004383. 10.1371/journal.pgen.1004383 24830394 PMC4022491

[36] Huang Q, Wang Y, Zhang L et al. Single-cell transcriptomics highlights immunological dysregulations of monocytes in the pathobiology of COPD. *Respir Res* 2022;23:367. 10.1186/s12931-022-02293-2 36539833 PMC9764587

[37] Hao Y, Stuart T, Kowalski MH et al. Dictionary learning for integrative, multimodal and scalable single-cell analysis. *Nat Biotechnol* 2024;42:293–304. 10.1038/s41587-023-01767-y 37231261 PMC10928517

[38] Knox C, Wilson M, Klinger CM et al. DrugBank 6.0: the DrugBank knowledgebase for 2024. *Nucleic Acids Res* 2024;52:D1265–75. 10.1093/nar/gkad976 37953279 PMC10767804

[39] Jumper J, Evans R, Pritzel A et al. Highly accurate protein structure prediction with AlphaFold. *Nature* 2021;596:583–9. 10.1038/s41586-021-03819-2 34265844 PMC8371605

[40] Volkamer A, Kuhn D, Rippmann F et al. DoGSiteScorer: a web server for automatic binding site prediction, analysis and druggability assessment. *Bioinformatics* 2012;28:2074–5. 10.1093/bioinformatics/bts310 22628523

[41] Kozakov D, Grove LE, Hall DR et al. The FTMap family of web servers for determining and characterizing ligand-binding hot spots of proteins. *Nat Protoc* 2015;10:733–55. 10.1038/nprot.2015.043 25855957 PMC4762777

[42] Szklarczyk D, Kirsch R, Koutrouli M et al. The STRING database in 2023: protein-protein association networks and functional enrichment analyses for any sequenced genome of interest. *Nucleic Acids Res* 2023;51:D638–46. 10.1093/nar/gkac1000 36370105 PMC9825434

[43] GTEx Consortium . The GTEx consortium atlas of genetic regulatory effects across human tissues. *Science* 2020;369:1318–30. 10.1126/science.aaz1776 32913098 PMC7737656

[44] Zhang X, Zhao H, Wan M et al. Associations of 2923 plasma proteins with incident inflammatory bowel disease in a prospective cohort study and genetic analysis. *Nat Commun* 2025;16:2813. 10.1038/s41467-025-57879-3 40118817 PMC11928603

[45] Pardiñas AF, Holmans P, Pocklington AJ et al. Common schizophrenia alleles are enriched in mutation-intolerant genes and in regions under strong background selection. *Nat Genet* 2018;50:381–9. 10.1038/s41588-018-0059-2 29483656 PMC5918692

[46] Gibson PG, Simpson JL. The overlap syndrome of asthma and COPD: what are its features and how important is it? *Thorax* 2009;64:728–35. 10.1136/thx.2008.108027 19638566

[47] Postma DS, Kerkhof M, Boezen HM et al. Asthma and chronic obstructive pulmonary disease: common genes, common environments? *Am J Respir Crit Care Med* 2011;183:1588–94. 10.1164/rccm.201011-1796PP 21297068

[48] Tu X, Donovan C, Kim RY et al. Asthma-COPD overlap: current understanding and the utility of experimental models. *Eur Respir Rev* 2021;30:190185. 10.1183/16000617.0185-2019 33597123 PMC9488725

[49] Davey Smith G, Hemani G. Mendelian randomization: genetic anchors for causal inference in epidemiological studies. *Hum Mol Genet* 2014;23:R89–98. 10.1093/hmg/ddu328 25064373 PMC4170722

[50] Zheng J, Baird D, Borges MC et al. Recent developments in Mendelian randomization studies. *Curr Epidemiol Rep* 2017;4:330–45. 10.1007/s40471-017-0128-6 29226067 PMC5711966

[51] Abdelaziz MH, Abdelwahab SF, Wan J et al. Alternatively activated macrophages; a double-edged sword in allergic asthma. *J Transl Med* 2020;18:58. 10.1186/s12967-020-02251-w 32024540 PMC7003359

[52] Wu J, Zhao X, Xiao C et al. The role of lung macrophages in chronic obstructive pulmonary disease. *Respir Med* 2022;205:107035. 10.1016/j.rmed.2022.107035 36343504

[53] Eapen MS, Hansbro PM, McAlinden K et al. Abnormal M1/M2 macrophage phenotype profiles in the small airway wall and lumen in smokers and chronic obstructive pulmonary disease (COPD). *Sci Rep* 2017;7:13392. 10.1038/s41598-017-13888-x 29042607 PMC5645352

[54] Wang J, Shiratori I, Uehori J et al. Neutrophil infiltration during inflammation is regulated by PILRα via modulation of integrin activation. *Nat Immunol* 2013;14:34–40. 10.1038/ni.2456 23142774

[55] Barnes PJ . Against the Dutch hypothesis: asthma and chronic obstructive pulmonary disease are distinct diseases. *Am J Respir Crit Care Med* 2006;174:240–3; discussion 243-244. 10.1164/rccm.2604008 16864717

[56] Revez JA, Bain LM, Watson RM et al. Effects of interleukin-6 receptor blockade on allergen-induced airway responses in mild asthmatics. *Clin Transl Immunology* 2019;8:e1044. 10.1002/cti2.1044 31223480 PMC6566140

[57] Tsicopoulos A, Chang Y, Ait Yahia S et al. Role of CCL18 in asthma and lung immunity. *Clin Exp Allergy* 2013;43:716–22. 10.1111/cea.12065 23786278

[58] Wang H, Pang J, Zhou Y et al. Identification of potential drug targets for allergic diseases from a genetic perspective: a mendelian randomization study. *Clin Transl Allergy* 2024;14:e12350. 10.1002/clt2.12350 38573314 PMC10994001

[59] Windhorst S, Song K, Gazdar AF. Inositol-1,4,5-trisphosphate 3-kinase-A (ITPKA) is frequently over-expressed and functions as an oncogene in several tumor types. *Biochem Pharmacol* 2017;137:1–9. 10.1016/j.bcp.2017.03.023 28377279 PMC5555585

[60] Shaw L, Wiedow O. Therapeutic potential of human elafin. *Biochem Soc Trans* 2011;39:1450–4. 10.1042/BST0391450 21936832

[61] de Serres F, Blanco I. Role of alpha-1 antitrypsin in human health and disease. *J Intern Med* 2014;276:311–35. 10.1111/joim.12239 24661570

[62] Defnet AE, Hasday JD, Shapiro P. Kinase inhibitors in the treatment of obstructive pulmonary diseases. *Curr Opin Pharmacol* 2020;51:11–8. 10.1016/j.coph.2020.03.005 32361678 PMC8504404

[63] Son S, Kim H, Lim H et al. CCN3/NOV promotes metastasis and tumor progression via GPNMB-induced EGFR activation in triple-negative breast cancer. *Cell Death Dis* 2023;14:81. 10.1038/s41419-023-05608-3 36737605 PMC9898537

[64] Betageri KR, Link PA, Haak AJ et al. The matricellular protein CCN3 supports lung endothelial homeostasis and function. *Am J Physiol Lung Cell Mol Physiol* 2023;324:L154–68. 10.1152/ajplung.00248.2022 36573684 PMC9925165

[65] Xiahou Z, Wang X, Shen J et al. NMI and IFP35 serve as proinflammatory DAMPs during cellular infection and injury. *Nat Commun* 2017;8:950. 10.1038/s41467-017-00930-9 29038465 PMC5643540

[66] Zeng J, Yang Z, Xu D et al. NMI functions as immuno-regulatory molecule in sepsis by regulating multiple Signaling pathways. *Inflammation* 2024;47:60–73. 10.1007/s10753-023-01893-4 37679586

[67] Chen L, Peters JE, Prins B et al. Systematic Mendelian randomization using the human plasma proteome to discover potential therapeutic targets for stroke. *Nat Commun* 2022;13:6143. 10.1038/s41467-022-33675-1 36253349 PMC9576777

[68] Daghlas I, Gill D. Mendelian randomization as a tool to inform drug development using human genetics. *Camb Prism Precis Med* 2023;1:e16. 10.1017/pcm.2023.5 38550933 PMC10953771

[69] Carter AR, Sanderson E, Hammerton G et al. Mendelian randomisation for mediation analysis: current methods and challenges for implementation. *Eur J Epidemiol* 2021;36:465–78. 10.1007/s10654-021-00757-1 33961203 PMC8159796

[70] Hizawa N . Clinical approaches towards asthma and chronic obstructive pulmonary disease based on the heterogeneity of disease pathogenesis. *Clin Exp Allergy* 2016;46:678–87. 10.1111/cea.12731 27009427

