# Supplementary material for: From early-onset asthma to chronic obstructive pulmonary disease: potential mediating proteins and therapeutic targets
Source: Brief Bioinform. 2026 May 6;27(3):bbag209. doi: 10.1093/bib/bbag209 (PMC13147455; doi:10.1093/bib/bbag209)
Supplement: Supplemental_Figures_bbag209 [file supplemental_figures_bbag209.docx]

**Figure S1. Predicted Ligand-Binding Pockets in CCN3 Identified by DoGSiteScorer.**


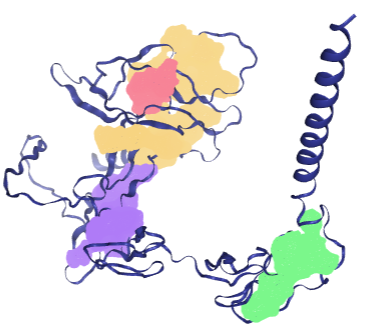


The CCN3 protein structure (drug score > 0.7) is shown in a ribbon representation. The predicted binding pockets are displayed as colored cavity surfaces, with green representing Pocket 2 (P_2), purple representing Pocket 1 (P_1), yellow representing Pocket 0 (P_0), and pink representing Pocket 3 (P_3). These correspond to the four highest-ranking pockets based on druggability scores.

**Figure S2. Predicted Ligand-Binding Hotspots of the NMI Protein Identified by FTMap.**


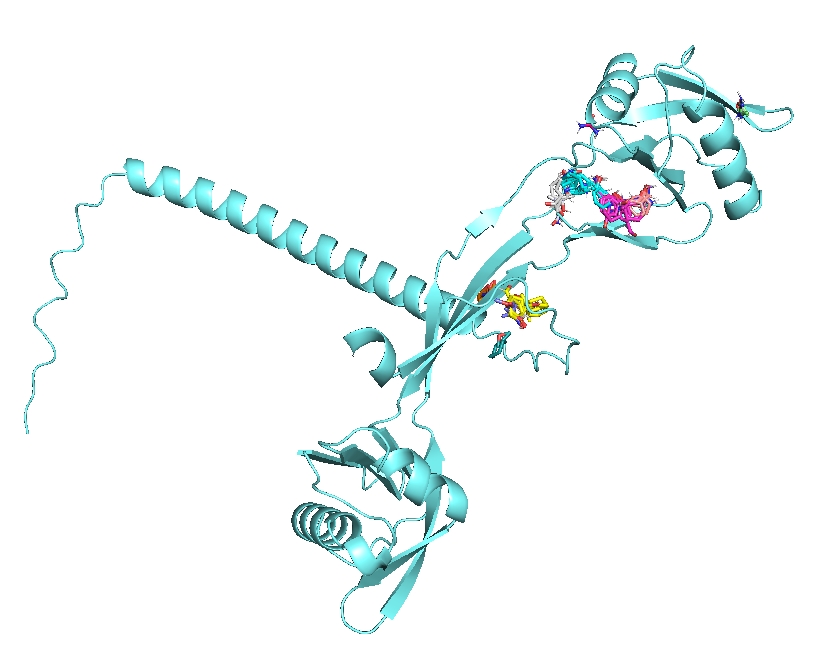


The overall structure of the NMI protein is shown in ribbon representation. Binding hotspots identified by FTMap are displayed as clusters of small-molecule probes in different colors. These clusters mark energetically favorable regions on the protein surface for small-molecule binding.

**Figure S3. Nonbonded Interaction Distribution of NMI Binding Hotspot Residues.**


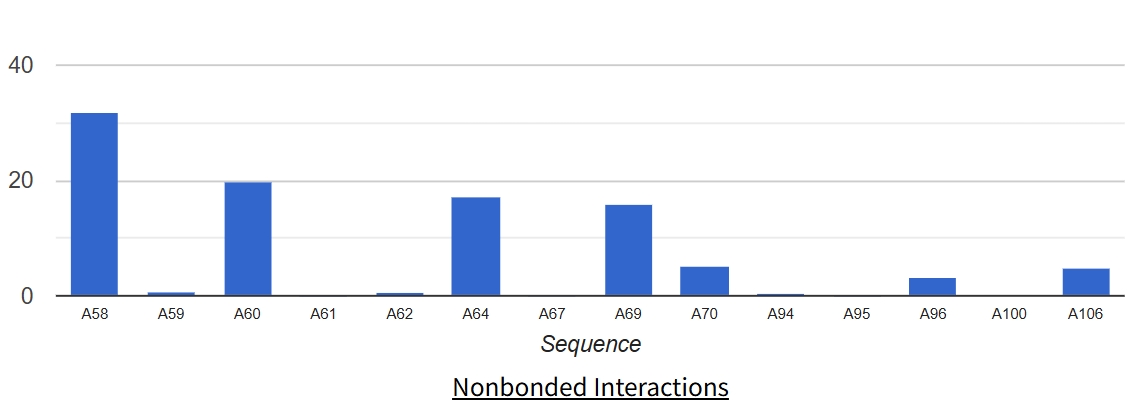


The x-axis represents the amino acid residue numbers in chain A of the NMI protein, and the y-axis shows the percentage contribution of each residue to non-bonded interactions. Residues with higher contribution percentages may play more important roles in maintaining protein structural stability or in potential small-molecule binding regions.

**Figure S4. Hydrogen-Bond Interaction Distribution of NMI Binding Hotspot Residues.**


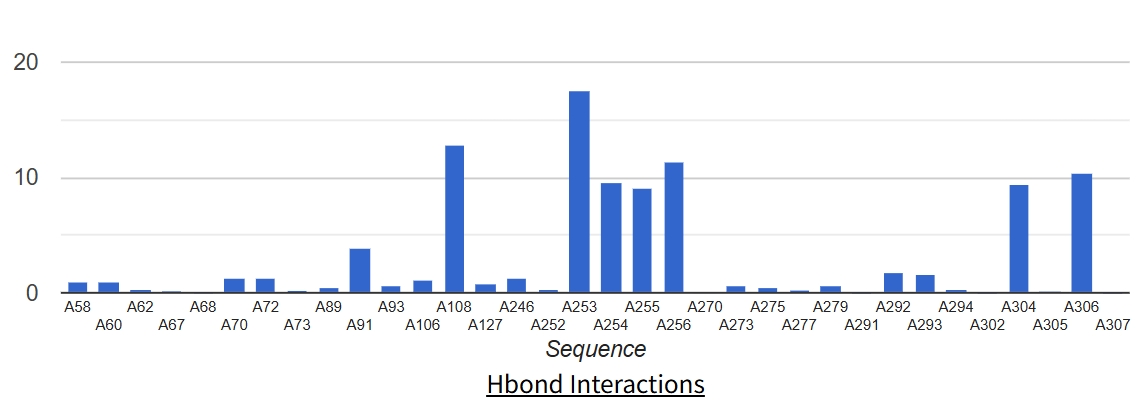


The x-axis represents the amino acid residue numbers in chain A of the NMI protein, and the y-axis shows the percentage contribution of each residue to hydrogen-bond interactions.

**Figure S5. Protein-Protein Interaction Network of NMI Derived from STRING.**


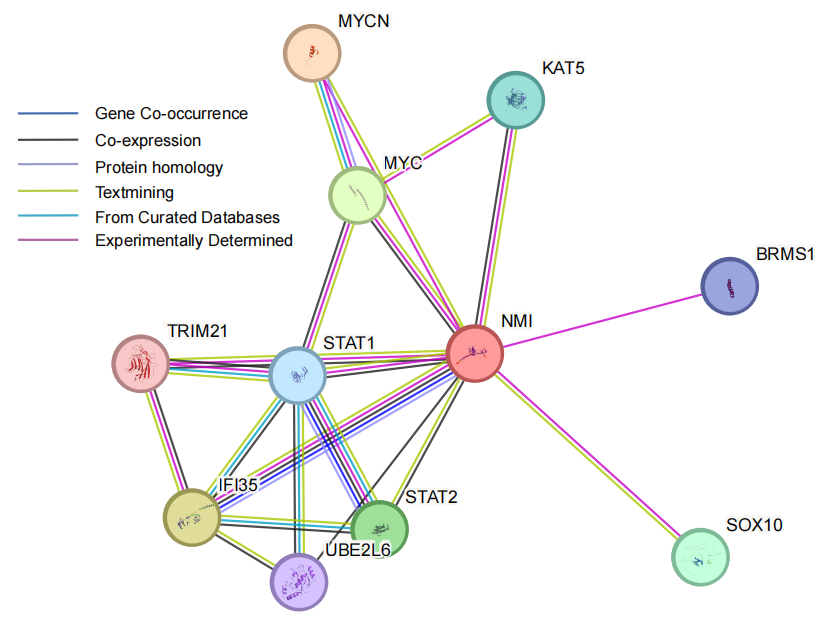


The network displays high-confidence interacting proteins of NMI (interaction score > 0.7) identified from the STRING database. Nodes represent proteins, and edges represent functional or physical associations supported by multiple evidence sources. Edge colors indicate the types of supporting evidence.
